# Cognitive perspective of osteoporosis among adults with type 2 diabetes mellitus: The Malaysian case

**DOI:** 10.1002/edm2.354

**Published:** 2022-06-10

**Authors:** Shaymaa Abdalwahed Abdulameer, Mohanad Naji Sahib, Syed Azhar Syed Sulaiman

**Affiliations:** ^1^ Faculty of Pharmacy Al‐Nisour University College Baghdad Iraq; ^2^ Clinical Pharmacy Department, School of Pharmaceutical Sciences Universiti Sains Malaysia Penang Malaysia

**Keywords:** osteoporosis, self efficacy, type 2 diabetes mellitus

## Abstract

**Introduction:**

Osteoporosis and diabetes are highly prevalent diseases. In addition, there is increasing evidence that diabetes is a common risk factor for decreasing bone mineral density and developing osteoporosis and fractures. Data on bone abnormalities in T2DM patients appear to be contradictory and complex, and the exact underlying mechanism is still unclear. Hence, the aims of this study were to assess cognitive perspective of osteoporosis among type 2 diabetes mellitus.

**Methods:**

An observational, cross‐sectional study design was chosen, where data were collected using a self‐report structured questionnaire including osteoporosis self‐efficacy (OSES‐M), knowledge Malay version. Quantitative ultrasound was used as prescreening tool for measuring bone health by applying T‐score.

**Results:**

The results showed that about 231 (51.30%) were males. The average age of the DM patients was 62.67 ± 9.24 years. Moreover, the majority of T2DM patient (343, 76.20%) had poor glycaemic control. The mean ± SD OSES‐M total score, OSES‐M Exercise subscale and OSES‐M Calcium subscale were 731.26 ± 209.83, 357.55 ± 121.23 and 373.71 ± 118.91, respectively. Overall, participants expressed a low self‐efficacy for both exercise and calcium intake experiences. In addition, a significant and positive correlation was found between OSES‐M and OKT‐M total scores (*n* = 450, *r*_s_ = 0.471, *p* < .05). Also, there were significant correlations between OKT‐M subscales and OSES‐M subscales. Furthermore, significant and positive correlations were found between T‐scores and OSES‐M total score (*r* = .191), OSES‐M Exercise subscale (*r* = .209) and OSES‐M Calcium subscale (*r* = .124). Moreover, significant associations, differences and correlations were found out between OSES‐M with many demographic and clinical data.

**Conclusion:**

Overall, participants expressed a low self‐efficacy for both exercise and calcium intake experiences. In addition, only 28.70% of the study population was found to have high OSES‐M level. Thus, self‐efficacy is important and effective determinants for gaining positive health behaviours towards osteoporosis.

## Graphical abstract

Type 2 diabetes mellitus (T2DM) and osteoporosis are both chronic conditions and the relationship between them is complex. However, both diseases require lifelong medical treatment and lifestyle adjustment with the main goal of preventing or minimizing their acute and chronic complications.

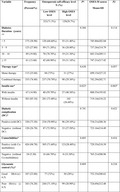

## 1 INTRODUCTION

Type 2 diabetes mellitus (T2DM) is one of the most rapidly growing, public health concerns with a major contributor to high levels of morbidity and mortality worldwide.^1^, ^2^ Over the last decades, the prevalence of T2DM has risen dramatically worldwide.^3^ In recent decades, continued urbanization and lifestyle modifications encourage sedentary lifestyles and increase the prevalence of overweight and obesity in Malaysia, which reflects the rise in diabetes prevalence over the last few decades.^4^ Moreover, recent study among Malaysian population showed large proportion of diabetic patients with poor or suboptimal glycaemic control.^5^

On the contrary, osteoporosis is a clinically silent, highly prevalent disease that has potentially devastating effects, which is largely preventable.^6^ It is widely considered as important worldwide public health issue, especially in postmenopausal and ageing population.^7^ Asian ancestry is considered a common risk factor for osteoporosis with compromised bone health compared to other ethnicities.^8^ Many studies showed that osteoporosis is underestimated, underdiagnosed and undertreated among the general ^9^ and diabetic population.^10^ Furthermore, there is increasing evidence that diabetes is a common risk factor for decreased bone mineral density (BMD) and developing osteoporosis and increased risk of fractures.^11^

Data on bone abnormalities in T2DM patients appear to be contradictory and complex, and the exact underlying mechanism is still unclear. While many studies support that diabetes increases fracture risk,^12^, ^13^ the relationships between T2DM and risk for bone loss have been inconsistent in other studies.^14^, ^15^ The Health Belief Model (HBM) can be used to postulate that optimal personal health behaviour change will be accomplish if they comprehend osteoprotective behaviours and self‐efficacy.^16^, ^17^ Hence, osteoporosis preventative lifestyle behaviour implementation has been shown to be valuable for bone health status.^18^ Whether an individual participates in osteoporosis preventative lifestyle behaviours or not, it is essential to have a primary understanding about their self‐efficacy regarding osteoporosis. Subsequently, health educational programmes can be planned and implemented accordingly.

To halt the progress of osteoporosis, assessment of osteoporosis self‐efficacy is required. Therefore, the study aimed to assess (1) the level of osteoporosis self‐efficacy Malay version (OSES‐M) among T2DM patients; (2) the correlation and differences of demographic characteristics and diabetes‐related data with osteoporosis self‐efficacy; (3) the correlation between osteoporosis self‐efficacy and knowledge scales and subscales; and (4) the correlation between T‐score measurement using quantitative ultrasound scan (QUS) and OSES‐M score.

## 2 MATERIALS AND METHODS

### 2.1 Study design and sampling method

An observational, cross‐sectional study design was chosen, where data were collected using a self‐report structured questionnaire among T2DM patients at Hospital Pulau Pinang (HPP) in Penang, Malaysia, from 1 August 2011 to 30 February 2012. In addition, the study included retrospective collection of clinical data from patients' medical records. The prevalence based sampling technique was used to identify the representative sample of T2DM patients. The T2DM prevalence in Malaysia is 14.90%.^19^, ^20^ Using an accepted margin of error of 5% and a 99% confidence interval with the addition of a 40% (to cover study drop‐outs), the target sample size was 474 patients. The inclusion criteria include the following: T2DM patients at least 2 years of disease duration, patients receiving oral hypoglycaemic agents with or without insulin for at least 1 year before inclusion in the study, age ≥30 years old, patients able to communicate in Bahasa Malaysia with no speech or hearing problems and patients willing to participate and given written informed consent. A convenient sampling method was used to select the study population.

### 2.2 Data collection tools

A structured questionnaire consisting of three parts was used; this included: (1) Personal socio‐demographic characteristics questionnaire and diabetes‐related data; (2) Osteoporosis Self‐Efficacy Scale Malay version (OSES‐M); and (3) Osteoporosis Knowledge test Malay version (OKT‐M). The Malay version of the OKT‐M and OSES‐M scales had been previously published and found to be reliable with an acceptable psychometric prosperity.^21^, ^22^ All participants were administered a questionnaire before they underwent a QUS examination using QUS (SONOST 3000) at the calcaneus.

### 2.3 Quantitative Ultrasound (QUS) measurements

Bone health measurements were carried out by a SONOST 3000 clinical bone densitometer (OsteoSys Co., Ltd.) at the calcaneus. Daily quality control was carried out on the ultrasound systems with phantom provided by the manufacturer. All the QUS measurements were performed by the same clinical pharmacist. Due to a lack of standardization in the field, we adopted World Health Organization (WHO) classification of bone health based on BMD T‐score as recommended by the manufacturer.^23^, ^24^

### 2.4 Ethical considerations

This study was approved by the Clinical Research Centre (CRC) of HPP and the Medical Research Ethics Committee (MREC) of the Ministry of Health, Malaysia, with ethic approval number: [^2^ dlm.KKM/NIHSEC/08/0804/P11‐101].

### 2.5 Statistical data analysis

Data were analysed using the computer program Predictive Analytics Software (PASW) for Windows version 19.0, and the level of statistical significance was set at *p* < .05 for all analyses. Percentages, frequencies, mean, median, standard deviations, chi‐square test, independent *t*‐test, one‐way analysis of variance (ANOVA) and Pearson and/or Spearman's correlations coefficient were used when necessary.

## 3 RESULTS

### 3.1 Demographic characteristics in relation to osteoporosis self‐efficacy

Four hundred and fifty subjects completed the study, after excluded 31 and 19 patients because lack of some clinical data or patients’ incomplete response, respectively. The results showed that about 231 (51.30%) were males. The average age of the DM patients was 62.67 ± 9.24 years. The majority of patients were as follows: not working (258; 57.30%), living in urban areas (360; 80%), obese (352, 78.20%) and had a monthly income less than RM 2000 (330, 73.30%). There was a significant relationship between OSES‐M levels and education groups, monthly income, family history of osteoporosis, family history of fracture and alcoholic habit (*p* < .05). Moreover, a significant difference in the total score of OSES‐M with the race, genders, education and alcoholic status was found (*p* < .05), as shown in Table 1.

**TABLE 1:** Relationships between osteoporosis self‐efficacy levels and patients' demographic characteristics (*N* = 450)

Variable	Frequency (Per cent%)	OSES‐M levels *N* (%)		*p* ^†^	OSES‐M scores Mean ± SD	*p* ^‡^
		Low OSES‐M level	High OSES‐M level			
		321 (71.3%)	129 (28.7%)			
Age groups						
<45 years	11 (2.40%)	10 (90.90%)	1 (9.10%)	.114	638.64 **±** 158.24	.529
45–54 years	78 (17.30%)	59 (75.60%)	19 (24.40%)		731.26 **±** 202.63	
55–64 years	166 (36.90%)	123 (74.10%)	43 (25.90%)		732.70 **±** 207.31	
≥65 years	195 (43.30%)	129 (66.20%)	66 (33.80%)		735.26 **±** 217.39	
Gender						
Male	231 (51.30%)	158 (68.40%)	73 (31.60%)	.157	752.93 **±** 215.794	.024^a^
Female	219 (48.70%)	163 (74.40%)	56 (25.60%)		708.41 **±** 201.328	
Race						
Malay	127 (28.20%)	91 (71.70%)	36 (28.30%)	.480	747.68 **±** 186.69	.011^a^
Chinese	204 (45.30%)	150 (73.50%)	54 (26.50%)		699.88 **±** 213.59	
Indian	119 (26.40%)	80 (67.20%)	39 (32.80%)		767.54 **±** 220.26	
Educational levels						
<12 years	285 (63.30%)	217 (76.10%)	68 (23.90%)	.003 ^b^	693.25 ± 208.44	<.001^c^
≥12 years	165 (36.70%)	104 (63%)	61 (37%)		796.92 ± 196.11	
Marital Status						
Single	70 (15.60%)	55 (78.60%)	15 (21.40%)	.145	697.71 **±** 208.81	.146
Not single	380 (84.40%)	266 (70%)	114 (30%)		737.44 ± 209.71	
Monthly income						
Less than RM 2000	330 (73.30%)	244 (73.90%)	86 (26.10%)	.043^a^	720.18 ± 201.98	.063
More than RM 2000	120 (26.70%)	77 (64.20%)	43 (35.80%)		761.73 ± 228.16	
Menopausal status (*N* = 219)						
Premenopausal	25 (11.40%)	21 (84%)	4 (16%)	.244	659.12 **±** 198.45	.194
Postmenopausal	194 (88.60%)	142 (73.20%)	52 (26.80%)		714.76 **±** 201.32	
Employment status						
Working	192 (42.70%)	133 (69.30%)	59 (30.70%)	.404	744.25 ± 223.19	.258
Not working	258 (57.30%)	188 (72.90%)	70 (27.10%)		721.60 ± 199.21	
Living place						
Rural	90 (20%)	57 (63.30%)	33 (36.70%)	.061	765.64 **±** 197.31	.082
Urban	360 (80%)	264 (73.30%)	96 (26.70%)		722.67 **±** 212.24	
Family history of osteoporosis						
No	392 (87.10%)	289 (73.70%)	103(26.30%)	.004^b^	723.99 **±** 209.58	.056
Yes	58 (12.90%)	32 (55.20%)	26 (44.80%)		780.41 **±** 206.69	
Family history of fracture						
No	359(79.80%)	265 (73.80%)	94 (26.20%)	.021^a^	723.71 **±** 206.64	.129
Yes	91 (20.20%)	56 (61.50%)	35 (38.50%)		761.07 **±** 220.65	
Smoking habit						
Not smoking	318 (70.70%)	232 (73%)	86 (27%)	.237	721.03 **±** 205.16	.108
Smoking	132 (29.30%)	89 (67.40%)	43 (32.60%)		755.92 **±** 219.53	
Alcohol habit						
Non alcoholic	356 (79.10%)	268 (75.30%)	88 (24.70%)	<.001^c^	714.63 **±** 209.15	.001^b^
Alcoholic	94 (20.90%)	53 (56.40%)	41 (43.60%)		794.24 **±** 201.32	
BMI (Kg/m^2^)						
Non‐obese (BMI ≤23 kg/m^2^)	98 (21.80%)	71 (72.40%)	27 (27.60%)	.782	745.41 **±** 198.04	.451
Obese(BMI >23 kg/m^2^)	352(78.20%)	250 (71%)	102 (29%)		727.32 **±** 213.10	

Abbreviations: BMI, body mass index; SD, standard deviation.

^†^ Association, chi‐square test, ^a^ *p* < .05, ^b^ *p* < .01, ^c^ *p* < .001.

^‡^ Difference.

### 3.2 Diabetes‐related variables in relation to osteoporosis self‐efficacy

Participants had a mean of diabetic duration of 8.65 ± 5.97 years. More than half of T2DM were on combination anti‐diabetic therapy (335, 74.40%) and only (67, 14.90%) use insulin. Regarding diabetic complication, 330 (73.30%) of patients had at least one diabetic complication and 236 patients (52.40%) had peripheral neuropathy. Moreover, the majority of T2DM patient (343, 76.20%) had poor glycaemic control. A significant association and difference between OSES‐M score and insulin use was found (*p* < .05), as shown in Table 2.

**TABLE 2:** Relationships between OSES‐M levels and diabetes‐related variables (*N* = 450)

Variable	Frequency (Per cent%)	Osteoporosis self‐efficacy level *N* (%)		*p* ^†^	OSES‐M scores Mean ± SD	*p* ^‡^
		Low OSES level	High OSES level			
		321 (71.3%)	129 (28.7%)			
Diabetes duration (years)^¶^						
<5	175 (38.90)	120 (68.60%)	55 (31.40%)	.366	745.80 **±** 202.04	.114
5–9	125 (27.80)	89 (71.20%)	36 (28.80%)		737.26 **±** 214.79	
10–14	89 (19.80)	70 (78.70%)	19 (21.30%)		683.33 **±** 209.11	
≥15	61 (13.60)	42 (68.90%)	19 (31.10%)		747.21 **±** 217.42	
Therapy type^§^						
Mono therapy	115 (25.60)	84 (73%)	31 (27%)	.638	699.15 **±** 227.22	.057
Combined therapy	335 (74.40)	237 (70.70%)	98 (29.30%)		742.29 **±** 202.71	
Insulin use^§^						
With insulin	67 (14.90)	40 (59.70%)	27 (40.30%)	.022^a^	800.55 **±** 195.02	.003^b^
Without insulin	383 (85.10)	281 (73.40%)	102 (26.60%)		719.14 **±** 210.22	
Diabetic complication (DC)^§^						
Positive (with DC)	330 (73.30)	234 (70.90%)	96 (29.10%)	.741	734.21 **±** 208.36	.622
Negative (without DC)	120 (26.70)	87 (72.50%)	33 (27.50%)		723.16 **±** 214.49	
Co‐morbidities^§^						
Positive (with Co‐morbidities)	426 (94.70)	305 (71.60%)	121(28.40%)	.603	729.35 **±** 210.39	.416
Negative (without Co‐morbidities)	24 (5.30)	16 (66.70%)	8 (33.30%)		765.21 **±** 200.96	
Glycaemic control (%)^§^						
Good HbA1c(<6.5)	107 (23.80)	77 (72%)	30 (28%)	.869	752.35 **±** 200.62	.234
Poor HbA1c (≥6.5)	343 (76.20)	244 (71.10%)	99 (28.90%)		724.69 **±** 212.48	

Abbreviation: SD, standard deviation.

^†^ Association, chi‐square test, ^a^ *p* < .05, ^b^ *p* < .01.

^‡^ Difference.

^§^ Independent *t*‐test.

^¶^ ANOVA.

### 3.3 Osteoporosis self‐efficacy assessment

The mean ± SD OSES‐M total score, OSES‐M Exercise subscale and OSES‐M Calcium subscale were 731.26 ± 209.83, 357.55 ± 121.23 and 373.71 ± 118.91, respectively. According to a cut‐off point, only 28.70% of the study population was found to have high OSES‐M level with an average score of 974.76 ± 87.14, while 71.30% were found to have low OSES‐M level with a mean score 633.41 ± 158.86. Overall, participants expressed a low self‐efficacy for both exercise and calcium intake experiences. Moreover, the mean ± SD of OKT total score, OKT‐M Exercise subscale and OKT‐M Calcium subscale were 12.55 ± 4.06, 8.60 ± 2.89 and 8.40 ± 3.36, respectively. Only 33.30% of the T2DM patients were found to have high level of osteoporosis knowledge.^22^

### 3.4 Correlations between osteoporosis self‐efficacy, knowledge total scores and subscale scores

Significant and positive correlations were found between OSES‐M and OKT‐M total scores (*n* = 450, *r*_s_ = .471, *p* < .05). Also, there were significant correlations between OKT‐M subscales and OSES‐M subscales. The results showed that OKT‐M‐Exercise was significantly and positively correlated with the OSES‐M Exercise (*n* = 450, *r*_s_ = .333, *p* < .05). In addition, the OKT‐M Calcium was significantly and positively correlated with the OSES‐M Calcium (*n* = 450, *r*_s_ = .412, *p* < .05).

### 3.5 Correlations between T‐scores and osteoporosis self‐efficacy (OSES‐M) and knowledge total scores and subscale scores

In this study, significant and positive correlations were found between T‐scores and OSES‐M total score (*r* = .191), OSES‐M Exercise subscale (*r* = .209) and OSES‐M Calcium subscale (*r* = .124) among T2DM patients (all *P*s <.05).

## 4 DISCUSSION

The OSES was selected to be used in the current study because of the fact that the OSES scale is one of the most widely used instruments that assess osteoporosis self‐efficacy.^25^ Self‐efficacy is the confidence of an individual ability to successfully organize and implement activities which are required to accomplish designated behaviours, despite various barriers and difficulties that might be faced in the future.^26^ Therefore, self‐efficacy is useful in understanding health behaviours. Research had revealed that individuals with high self‐efficacy prefer to do more challenging activities than those with low self‐efficacy and changing to a healthier lifestyle behaviour is related to their knowledge, health beliefs and self‐efficacy.^27^ In addition, the most accepted source of self‐efficacy accumulation was the previous successful experiences of individuals in implementing the desired behaviours.^28^

By applying the cut‐off value (858) of validated Malaysian version (OSES‐M),^21^ more than 70% of T2DM patients had a low level of OSES‐M. In this study, the confidence percent of OSES‐M was 60.94%, which was comparable to other studies.^29^, ^30^ Overall, participants expressed a low self‐efficacy for both exercise and calcium intake experiences. In addition, the mean confidence score for the OSES‐M Calcium intake subscale was reported to be higher than OSES‐M Exercise subscale (62.28% and 59.59%, respectively). Similarly, many studies have shown that OSES‐Calcium subscale was higher than the OSES‐Exercise subscale.^30^, ^31^ In contrast, other studies have shown that OSES‐Exercise subscale was higher than the OSES‐Calcium subscale or both subscales were low.^32^, ^33^, ^34^ Therefore, increasing the awareness of T2DM patients and as a consequence self‐efficacy perceptions towards osteoporosis are of significant importance in osteoporosis preventive behaviours.^35^

In general, lower health status is associated with low socioeconomic status.^36^, ^37^ This fact supports the current study results, which showed that self‐efficacy was more affected by socioeconomic factors such as education, gender, ethnicity and monthly income, but not with age. In the current study, there were significant relationship and difference between the two levels of OSES‐M and education groups (Table 1). In contrast, two studies have shown insignificant differences between the self‐efficacy towards osteoporosis and educational levels of the patients.^29^, ^38^ However, it was known that educational level may help to acquire knowledge, belief, motivation and self‐efficacy to healthier behaviours.^39^ Previous studies showed that osteoporosis preventive education programmes significantly increased osteoporosis self‐efficacy scores.^40^, ^41^ Thus, the assessment of self‐efficacy can help to estimate osteoporosis prevention behaviour and creates the need and opportunity for motivational and educational efforts targeting the high‐risk population.

In addition, a significant relationship between the two OSES‐M levels and monthly income was found (Table 1). This can be interpreted as the fact that low income inevitably affected the patients from developing healthier behaviours regarding nutritional choices and exercise behaviours which are necessary at all stages of life to prevent osteoporosis. In contrast, a study of Turkish women has shown insignificant differences between the income levels and their self‐efficacy scores.^29^ A significant association and difference between OSES‐M score and insulin use was found. Intuitively, there were no direct relationships between the diabetes‐related variables and osteoporosis self‐efficacy. However, indirect relation may explain this result. In this study, the results showed a higher proportion of high OSES‐M level found among patients with insulin use. This may give an indication regarding the information provided by the healthcare professionals to diabetic patients, which may involve a broad range of advice about diabetes and its complications (diabetes is one of the risk factors of osteoporosis).

According to various health promotion models, a person's knowledge towards potential health problems was expected to influence and encourage the person to engage in self‐care health behaviours when mediated by health belief and self‐efficacy.^42^ In addition, research demonstrated that osteoporosis knowledge was a preparation stage for initiating and continuing positive health behaviours to prevent the illness, which was an important determinant for the self‐efficacy.^43^ Accordingly, many studies demonstrated a significant positive correlation between osteoporosis knowledge and osteoporosis self‐efficacy, as this study.^29^, ^44^ It had been determined that adequate knowledge of exercise and calcium intake is a strong determinant of participant's self‐efficacy to engage in healthier behaviours.^29^, ^45^ Therefore, an educational programme to increase the knowledge and subsequent perceived benefits of exercise are necessary to improve the self‐efficacy towards osteoporosis among T2DM patients.

A well‐developed educational program is essential for improving osteoporosis outcome by increasing bone mass and decreasing fracture, as well as improving diabetes outcome (such as glycaemic control). Hence, evaluation of patient's educational needs is a vital first step, not only for osteoporosis, but also for improving T2DM patient outcomes and reducing the risk of long‐term complications.

Although these interesting results, every study has a limitations. The study limitations were using convenient sampling, cross‐sectional design and only targeted outpatients type 2 diabetes mellitus, who may further limits the generalization of the findings to the entire Malaysian population. In addition, there was a possibility of under‐ or over‐reporting of osteoporosis self‐efficacy as they were self‐reported variables and the QUS method was used in this study as an alternative in the evaluation of bone status for osteoporosis screening.

## 5 CONCLUSIONS

The study finding clearly indicated that more than 70% of the sample population had a low osteoporosis self‐efficacy level. Overall, participants expressed a low self‐efficacy for both exercise and calcium intake experiences and their confidence for performing all listed behaviours were low. The results of this study were of great importance as it specified the factors that predict osteoporotic conditions and help in initiating osteoporosis preventive behaviours. As self‐efficacy is important in understanding health behaviour, it is necessary to increase the self‐efficacy perceptions towards osteoporosis preventive behaviours.

## AUTHOR CONTRIBUTIONS

**Shaymaa Abdalwahed Abdulameer:** Conceptualization (equal); data curation (equal); formal analysis (equal); investigation (equal); methodology (equal); project administration (equal); validation (equal); writing – original draft (equal); writing – review and editing (equal). **Mohanad Naji Sahib:** Data curation (equal); formal analysis (equal); methodology (equal); validation (equal); writing – original draft (equal). **Syed Azhar Syed Sulaiman:** Supervision (lead); validation (equal); writing – original draft (equal).

## CONFLICT OF INTEREST

No author has any conflict of interest.

### ETHICAL APPROVAL

Approval of the research protocol: The study was approved by the Clinical Research Centre (CRC) of Hospital Pulau Pinang and the Medical Research Ethics Committee (MREC) of the Ministry of Health, Malaysia and conducted in accordance with the Declaration of Helsinki (October 2011).

Informed consent: All subjects gave their written informed consent for inclusion before they participated in the study.

Approval date of Registry and Registration No. of the study: [(2) dlm.KKM/NIHSEC/08/0804/P11‐101], Registration ID: NMRR‐11‐28‐8209 on 28 March 2011.

Animal Studies: N/A.

## Data Availability

The data that support the findings of this study are available from the corresponding author upon reasonable request.
